# Association of HLA-G 3′ UTR polymorphism and expression with the progression of cervical lesions in human papillomavirus 18 infections

**DOI:** 10.1186/s13027-018-0217-2

**Published:** 2018-12-29

**Authors:** Hui-Hui Xu, Xia Zhang, Hai-Hong Zheng, Qiu-Yue Han, Ai-Fen Lin, Wei-Hua Yan

**Affiliations:** 1 Medical Research Center, Taizhou Hospital of Zhejiang Province, Wenzhou Medical University, Linhai, Zhejiang, China; 2 Laboratory of Gynecologic Oncology, Taizhou Hospital of Zhejiang Province, Wenzhou Medical University, Linhai, Zhejiang, China; 3 Human Tissue Bank, Taizhou Hospital of Zhejiang Province, Wenzhou Medical University, Linhai, Zhejiang, China; 4 Department of Pathology, Taizhou Hospital of Zhejiang Province, Wenzhou Medical University, Linhai, Zhejiang, China

**Keywords:** Human leukocyte antigen-G, Polymorphism, Expression, Human papillomavirus 18, Precancerous cervical lesions, Cervical cancer

## Abstract

**Background:**

Human leukocyte antigen (HLA)-G is an immune checkpoint molecule, which expression in cervical cancer cells enables them to escape immunosurveillance. To date, limited information has been published on the association of HLA-G genetic background in malignant cells with levels of HLA-G expression and the clinical outcome of patients.

**Methods:**

We investigated the influence of the HLA-G *14 bp In/Del* (rs66554220) and + 3142*C/G* (rs1063320) polymorphisms in 130 cases of HPV16 infection, 130 cases of HPV18 infection and 185 age-matched, unrelated, HPV-negative, and cytologically normal Chinese Han women. Case-matched cervical biopsy tissues were evaluated by immunohistochemistry.

**Results:**

Our findings show that the frequency of alleles, *14 bp In* (38.5% vs 29.2%, OR = 1.52, *P* < 0.05) and + 3142*G* (72.7% vs 57.0%, OR = 2.01, *P* < 0.05), were significantly increased in the HPV18-infected group compared with the control group. The *HLA-G* polymorphisms (alleles *14 bp In* and + 3142*G*) are also associated with the progression of HPV18-related cervical lesions. Moreover, HLA-G expression increased from CIN1 to CIN2/3 lesions and was highest in patients with adenocarcinoma; however, a significant association between these characteristics and the HLA-G polymorphisms was not observed.

**Conclusion:**

Our results support that the *HLA-G 14 bp In* and + 3142*G* alleles are risk factors for HPV18 infections and influence the progression of HPV18-related cervical lesions. This suggests that HLA-G-driven immune mechanisms play an important role in cervical carcinogenesis.

## Introduction

Cervical cancer is the second most common cancer, as well as the third leading cause of cancer mortality among women, worldwide [1]. Among all possible etiological factors, an infection with high-risk human papillomavirus (hrHPV) is necessary for the development of high-grade cervical intraepithelial neoplasia (CIN2/3) and invasive cervical cancer (ICC), but it is not sufficient for these conditions [2]. HPV16 and HPV18 are the two most carcinogenic HPV genotypes, accounting for 55–60% and 10–15% of ICC worldwide, respectively. However, only a small proportion of women infected with HPV develop CIN2/3 (10% of HPV infections) and cervical cancer (less than 1% of HPV infections); in most women, the infection regresses spontaneously. Evidence suggests that other factors, including the immune system and genetics of the host, as well as the viral genotype, appear to play a role in the development of cervical cancer [3].

Human leukocyte antigen-G (HLA-G) is a non-classical HLA class I molecule with well-characterized immunomodulatory activities, including downregulation of the innate and adaptive immune responses and the induction of tolerance. HLA-G interacts with the inhibitory receptors, immunoglobulin-like transcript (ILT)-2, ILT-4, and KIR2DL4 through several mechanisms. These mechanisms include preventing the cytotoxic activity of natural killer and cytotoxic T cells, CD4^+^ T cell alloproliferative responses and the maturation and function of antigen-presenting cells. Additionally, HLA-G may exert long-term tolerogenic effects by modulating cytokine production and inducing immune regulatory cells [4]. Under various pathological conditions, HLA-G expression has been reported to be involved in tumor cell immune escape, transplantation, viral infection, inflammation, and autoimmune diseases [5]. HLA-G mRNA can be generated in seven different isoforms due to the alternative splicing activity of its primary transcripts, where HLA-G1, −G2, −G3, and HLA-G4 are membrane-bound, and HLA-G5, −G6, and HLA-G7 are soluble isoforms. HLA-G exhibits low polymorphism levels, with only 56 alleles and 18 proteins described so far (http://hla.alleles.org/nomenclature/stats.html). The *14 bp In/Del* polymorphism (rs66554220) is located at the 3′ untranslated region (3′UTR) and has been proven to influence the stability and splicing patterns of *HLA-G* mRNA, which is associated with HLA-G expression status [6, 7]. The SNP, + 3142*C/G* (rs1063320), which is known to be within a predicted binding site for microRNAs, is thought to be important in facilitating HLA-G expression through post-transcriptional regulation [8].

Our previous findings indicated that HLA-G expression was associated with disease progression in cervical cancer [9]. Recently, we reported that *HLA-G 3′ UTR* polymorphisms were genetic susceptibility factors for active HPV infections, especially for high-risk HPV infections [10]. Whether the expression of HLA-G depends on the HPV type, the genetic background of patients or both is a question that has not yet been answered. The aim of this study was to perform a paired analysis of the relationship between *HLA-G 3′ UTR* polymorphisms (*14 bp In/Del* and + 3142*C/G*) and HLA-G expression in patients with HPV16 or HPV18 infections (stratified according to lesion severity).

## Materials and methods

### Ethics statement

This study was approved by the Institutional Medical Ethics Review Board of Taizhou Hospital in Zhejiang Province. Biosamples were provided by the Tissue Bank of Taizhou Hospital in Zhejiang Province (National human genetic resources sharing service platform of China 2005DKA21300). All of the participants were Chinese Han women, who provided written, informed consent to participate in the study. Confidentiality was ensured during the data collection process, which was completed by Taizhou Hospital; data were analyzed anonymously.

### Study design and subjects

The subjects of this study were women who underwent cervical cancer screening in the gynecological clinic at Taizhou Hospital of Zhejiang Province. Of the 17,669 women, 3210 (18.2%) were hrHPV positive. In the hrHPV group, HPV52 was the most prevalent genotype (871, 27.1%), followed by HPV16 (545, 17.0%), HPV58 (483, 15.0%), HPV39 (278, 8.7%), HPV18 (264, 8.2%), and HPV56 (257, 8.0%). These results were previously reported in our TZHPV study [11]. The inclusion criteria of this study encompassed the following: women with an HPV16 or HPV18 single infection, no current pregnancies, and no history of total uterus or cervix resection.

A total of 445 women between the ages of 18 to 72 years were recruited and included 130 cases of HPV16 infection, 130 cases of HPV18 infection and 185 cases involving those who were HPV negative and cytologically normal. According to the 2012 ASCCP consensus guidelines for the abnormal cervical cancer screening, women with HPV16 or HPV18 positive should be referred directly to colposcopy. Finally, 153 case-matched cervical biopsy tissues were collected from patients harboring HPV16 or HPV18 infections. The clinical characteristics of the study population are shown in Table 1. Cervical exfoliated cell sample (obtained through cervical scraping) was collected from all eligible women. Biopsy tissues were sectioned and histologically evaluated following hematoxylin and eosin staining (H&E) by two experienced histopathologists, in a double-blind protocol.

**Table 1:** Study Participant Characteristics

Characteristic	Controls	HPV16 group	HPV18 group
	(*n* = 185)	(*n* = 130)	(*n* = 130)
Age at screening, years			
Median	40.8 ± 8.0	41.3 ± 10.0	41.0 ± 9.8
Range	18–69	20–71	21–72
HPV status			
HPV negative	185	0	0
HPV16 positive	0	130	0
HPV18 positive	0	0	130
Cytology			
NILM	185	40(52.6)	89(88.1)
ASCUS	0	10(13.2)	5(5.0)
ASC-H	0	11(14.5)	4(4.0)
LSIL	0	12(15.8)	3(3.0)
HSIL	0	3(3.9)	0(0.0)
Histology diagnosis			
Normal/Cervicitis	–	24(27.9)	50(74.6)
CIN1	–	10(11.6)	11(16.4)
CIN2	–	15(17.4)	1(1.5)
CIN3	–	31(36.0)	3(4.5)
SCC	–	6(7.0)	0(0.0)
ADC	–	0(0.0)	2(3.0)

*Abbreviations*, *HPV* human papillomavirus, *NILM* negative for intraepithelial lesion or malignancy, *ASCUS* atypical squamous cells of undetermined significance, *ASC-H* atypical squamous cells and cannot exclude high-grade squamous intraepithelial lesions, *LSIL* low-grade squamous intraepithelial lesions, *HSIL* high-grade squamous intraepithelial lesions, *CIN* cervical intraepithelial neoplasia, *SCC* squamous cell carcinoma, *ADC* adenocarcinoma

### HPV genotyping and sequencing

HPV genotyping was performed according to a previously described protocol, using a commercial detection kit purchased from Tellgen Life Science (Shanghai, China), which was approved by the China Food and Drug Administration (CFDA Certified NO. (2014): 3400847) [11]. Briefly, the protocol is based on the GP5+/bioGP6 + -PCR, and involves using sets of biotinylated amplimers and a multiplex human papillomavirus genotyping (MPG) methods with bead-based Luminex suspension array technology [11, 12], which is able to simultaneously identify 14 high-risk HPV types, 12 low-risk HPV types and β-globin gene (internal control).

The conserved *L1* regions of HPV 16 and HPV 18 isolates were amplified from either HPV16 or HPV18-positive samples using consensus primers MY09/11(455 bp). The PCR reactions were pre-heated for 5 min at 95 °C, followed by 40 repeated cycles of 94 °C for 30 s, 55 °C for 30 s, 72 °C for 30 s, and a final extension step at 72 °C for 7 min. The PCR products were visualized using 1.5% agarose gels and were then sent to the Beijing Genomics Institute for DNA sequencing (ABI 3730, Applied Biosystems, Foster City, CA, USA).

### HLA-G genotyping

Genomic DNA was extracted using a QIAGEN kit (QIAGEN, Grand Island, NY), according to the manufacturer’s recommendations. The PCR analyses of the *HLA-G 14 bp In/Del* and + 3142*C/G* polymorphisms were performed using a standard protocol, as previously described [10]. Briefly, for each sample, a double PCR reaction was performed to verify the presence of the alleles + 3142C or + 3142G. Moreover, we performed another PCR reaction with the primers, 14F-5′-GTTGAGGGGAACAGG GGACAT-3′ and 14R-5′-AAAGTTCTCATGTCTTCCATTT-3′, to confirm the *14 bp In/Del* polymorphism in each sample. Globin signals were amplified in parallel with each sample, and used as internal controls. The amplified fragments were visualized by 10%, non-denaturing, polyacrylamide gel electrophoresis (PAGE), stained with ethidium bromide and scored by three different observers. The PCR products were confirmed through sequencing (ABI 3730, Applied Biosystems, Foster City, CA, USA).

### Immunohistochemistry and staining evaluation

Immunohistochemistry (IHC) was performed following a standard protocol, as previously reported [9]. In the staining procedure, the guidelines of the Dako EnVision kit (Dako, Glostrup, Denmark), were strictly followed. The monoclonal antibody, 4H84 (able to detect all HLA-G isoforms), was purchased from Exbio (Prague, Czech Republic). The percentage of HLA-G expression was considered to be positive at > 5% and negative at ≤5% HLA-G staining, in cervical lesions. Cells were assigned a value (positive or negative) based on the presence or absence of HLA-G staining, irrespective of staining intensity [13].

### Statistical analysis

A statistical analysis was performed using SPSS 16.0 software (SPSS Inc., Chicago, IL). Chi-squared and Fisher’s exact tests were used to compare the *HLA-G* allelic and genotypic frequencies of controls and patients. They were also used to evaluate the relative CIN2 or CIN2+ (more severe) risk associated with HPV genotypes, odds ratios (OR) and the relative 95% confidence interval (CI). An analysis of covariance (ANCOVA) was used to adjust the mean age between the HPV18 subgroups. *P* values were two-sided, and the results were considered statistically significant if the *P* value was 0.05 or less.

## Results

### Characteristics of the population

One hundred and thirty patients infected with HPV16 were successfully sequenced for the *L1* gene and classified as the HPV16 group, 130 patients infected with HPV18 (confirmed by sequencing for the *L1* gene) were classified as the HPV18 group, and 185 women without an HPV infection and exhibiting normal cervical cytology, were classified as the control group. Eighty-six HPV16-infected patients were diagnosed via cervical biopsy, including 24 normal cases/cervicitis (27.9%), 10 CIN1 (11.6%), and 52 CIN2+ (60.5%). Sixty-seven HPV18-infected patients were diagnosed via cervical biopsy, including 50 normal cases/cervicitis (74.6%), 11 CIN1 cases (16.4%), and 6 CIN2+ cases (9.0%). Compared with HPV18, the OR for CIN2+ in HPV16-infected patients was 15.5 (95%CI, 6.8–35.6, *P* < 0.001), which indicated that women infected with HPV16 may be at an increased risk for CIN1 lesions. These lesions may progress to CIN2 or a more deleterious lesion. HPV18 may have a lower oncogenic potential or require a longer period of time to become carcinogenic [14]. However, HPV18 has a greater tendency to cause adenocarcinoma (ADC) in comparison to HPV16.

### HLA-G 3′ UTR polymorphism association with HPV18 infections

In this study, all of the genotypes fit the Hardy–Weinberg equilibrium expectations (data not shown). The frequency of the *14 bp In/Del* polymorphism was 31.9 and 68.1% in the HPV16 group; 38.5 and 61.5% in the HPV18 group; and 29.2 and 70.8% in the control group, respectively. We found that the *14 bp In/Del* alleles and genotypes were not associated with a susceptibility to HPV16 infections. However, the frequency of the allele, *14 bp Del*, was significantly decreased in the HPV18 group, compared with the control group (61.5% vs 70.8%, OR = 0.66; *P* = 0.015); similar significance was observed for the genotype, *Del*/*Del* (35.4% vs 48.7%, OR = 0.58, *P* = 0.019) (Table 2).

**Table 2:** Distribution of HLA-G allele, genotype and haplotype in controls and patients, stratified according to HPV type

	Control group	Patient group			
		HPV 16 group		HPV 18 group	
Alleles	**n (%)**	***n*** **(%)**	OR(95% CI)	***n*** **(%)**	OR(95% CI)
14 bp In/Del					
*In*	108(29.2)	83(31.9)	1.14(0.81–1.60)	100(38.5)	**1.52(1.08–2.12)** ^**a**^
*Del*	262(70.8)	177(68.1)	0.88(0.62–1.24)	160(61.5)	**0.66(0.47–0.92)** ^**a**^
*n*	370	260		260	
+3142 C/G					
*C*	159(43.0)	109(41.9)	0.96(0.69–1.32)	71(27.3)	**0.50(0.36–0.70)** ^**b**^
*G*	211(57.0)	151(58.1)	1.04(0.75–1.44)	189(72.7)	**2.01(1.43–2.82)** ^**b**^
*n*	370	260		260	
Genotypes					
14 bp In/Del					
*In/In*	13(7.0)	13(10.0)	1.47(0.66–3.28)	16(12.3)	1.86(0.87–3.98)
*In/Del*	82 (44.3)	57(43.8)	0.98(0.62–1.54)	68(52.3)	1.38(0.88–2.16)
*Del/Del*	90(48.7)	60(46.2)	0.90(0.57–1.42)	46(35.4)	**0.58(0.36–0.92)** ^**c**^
*n*	185	130		130	
+3142 C/G					
*C/C*	39(21.1)	25(19.2)	0.89(0.51–1.56)	8(6.2)	**0.25(0.12–0.52)** ^**d**^
*C/G*	81(43.8)	59(45.4)	1.07(0.68–1.68)	55(42.3)	0.94(0.60–1.48)
*G/G*	65(35.1)	46(35.4)	1.01(0.63–1.62)	67(51.5)	**1.96(1.24–3.10)** ^**e**^
*n*	185	130		130	
Haplotypes					
*In/G*	108(29.2)	83(31.9)	1.14(0.81–1.60)	100(38.5)	**1.52(1.08–2.12)** ^**f**^
*Del/G*	103(27.8)	68(26.2)	0.92(0.64–1.31)	89(34.2)	1.35(0.96–1.90)
*Del/C*	159(43.0)	109(41.9)	0.96(0.69–1.32)	71(27.3)	**0.50(0.35–0.70)** ^**g**^
*n*	370	260		260	

Significant association is highlighted in bold

*CI* confidence interval, *OR* odds ratio

^a^χ^2^ = 5.94, *P* = 0.015

^b^χ^2^ = 16.17, *P* = 0.000

^c^χ^2^ = 5.48, *P* = 0.019

^d^χ^2^ = 13.40, *P* = 0.000

^e^χ^2^ = 8.44, *P* = 0.004

^f^χ^2^ = 5.94, *P* = 0.015

^g^χ^2^ = 16.17, *P* = 0.000

Moreover, the frequency of the + 3142*C/G* polymorphism was 41.9 and 58.1% in the HPV16 group; 27.3 and 72.7% in the HPV18 group; and 43.0 and 57.0% in control group, respectively. We found that the + 3142*C/G* alleles and genotypes were not associated with a susceptibility to HPV16 infections. However, the frequency of the allele, + 3142*G*, was significantly increased in the HPV18 group, compared with the control group (72.7% vs 57.0%, OR = 2.01, *P* < 0.001); similar significance was observed for the genotype, + 3142*G/G* (51.5% vs 35.1%, OR = 1.96, *P* = 0.004). Furthermore, the frequency of the haplotype, *14 bp Del/*+3142*C*, was also significantly decreased in the HPV18 group (27.3% vs 43.0%, OR = 0.50, *P* < 0.001) (Table 2). These results show that the *HLA-G* polymorphism (alleles *14 bp In* and + 3142*G*) is a susceptibility factor for HPV18 infections.

### HLA-G 3′ UTR polymorphism association with the progression of HPV18-related cervical lesions

To test the effect of the HLA-G polymorphism on the progression of cervical lesions from CIN1 to cervical cancer in the HPV18 group, we carried out a separate analysis restricted to normal histology, CIN1 and CIN2+. When compared with the control group, the frequency was dramatically increased in CIN1 subgroup for the allele, +3142*G* (81.8% vs 57.0%, OR = 3.39, 95% CI: 1.19–9.65, *P* = 0.022), and the genotype, +3142*G/G* (63.6% vs 35.1%, OR = 3.23, 95%CI: 0.96–10.83, *P* = 0.056). Furthermore, we found that the frequency of the haplotype, *14 bp Del/*+3142*C*, was significantly decreased in CIN1 (18.1% vs 43.0%, OR = 0.29, 95%CI: 0.10–0.84, *P* = 0.022). When the CIN2+ subgroup was compared with the control group, the frequency of the genotype, *14 bp In/In*, was also significantly increased (33.3% vs 7.0%, OR = 6.62, *P* = 0.018) (Table 3). These results show that the *HLA-G* polymorphism (alleles *14 bp In* and + 3142*G*) is associated with the progression of HPV18-related cervical lesions.

**Table 3:** Distribution of HLA-G allele, genotype and haplotype by disease status

	Control group	HPV 18 group					
		Normal		CIN 1		CIN 2+	
	40.8 ± 8.0	39.1 ± 8.9		40.5 ± 10.1		50.2 ± 12.3	
Alleles	***n*** **(%)**	***n*** **(%)**	OR(95% CI)	***n*** **(%)**	OR(95% CI)	***n*** **(%)**	OR(95% CI)
*14 bp In/Del*							
+ 14 bp	108(29.2)	38(38.0)	1.48(0.94–2.36)	8(36.4)	1.39(0.57–3.39)	6(50.0)	2.43(0.79–7.45)
− 14 bp	262(70.8)	62(62.0)	0.67(0.42–1.07)	14(63.6)	0.72(0.29–1.77)	6(50.0)	0.41(0.13–1.27)
*n*	370	100		22		12	
*+3142 C/G*							
C	159(43.0)	23(23.0)	**0.40(0.24–0.65)** ^**a**^	4(18.2)	**0.29(0.10–0.84)** ^**b**^	4(33.3)	0.66(0.20–2.23)
G	211(57.0)	77(77.0)	**2.52(1.53–4.16)** ^**a**^	18(81.8)	**3.39(1.19–9.65)** ^**b**^	8(66.7)	1.51(0.45–5.06)
*n*	370	100		22		12	
Genotypes							
*14 bp In/Del*							
+ 14 bp/+ 14 bp	13(7.0)	5(10.0)	1.47(0.50–4.33)	1(9.1)	1.32(0.16–11.14)	2(33.3)	**6.62(1.37–31.40)** ^**c**^
+ 14 bp/−14 bp	82(44.3)	28(56.0)	1.60(0.85–2.99)	6(54.5)	1.51(0.45–5.09)	3(50.0)	1.26(0.25–6.39)
− 14 bp/−14 bp	90(48.7)	17(34.0)	0.54(0.28–1.04)	4(36.4)	0.60(0.17–2.11)	1(16.7)	0.21(0.03–1.53)
*n*	185	50		11		6	
*+ 3142 C/G*							
C/C	39(21.1)	1(2.0)	**0.08(0.02–0.37)** ^**d**^	0(0.0)	0(0.00–0.00)	1(16.7)	0.75(0.09–6.59)
C/G	81(43.8)	21(42.0)	0.93(0.49–1.75)	4(36.4)	0.73(0.21–2.59)	2(33.3)	0.64(0.12–3.56)
G/G	65(35.1)	28(56.0)	**2.35(1.26–4.40)** ^**e**^	7(63.6)	3.23(0.96–10.83)	3(50.0)	1.85(0.37–9.24)
*n*	185	50		11		6	
*Haplotypes*							
+ 14 bp /G	108(29.2)	38(38.0)	1.49(0.94–2.36)	8(36.4)	1.39(0.57–3.39)	6(50.0)	2.43(0.79–7.45)
− 14 bp /G	103(27.8)	39(39.0)	**1.66(1.05–2.62)** ^**f**^	10(45.5)	2.16(0.92–5.07)	2(16.7)	0.52(0.11–2.35)
− 14 bp /C	159(43.0)	23(23.0)	**0.40(0.24–0.65)** ^**g**^	4(18.1)	**0.29(0.10–0.84)** ^**h**^	4(33.3)	0.66(0.20–2.23)
*n*	370	100		22		12	

Significant association are highlighted in bold

*CI* confidence interval, *OR* odds ratio

^a^χ^2^ = 13.23, *P* = 0.000

^b^χ^2^ = 5.25, *P* = 0.022

^c^χ^2^ = 5.53, *P* = 0.018

^d^χ^2^ = 10.15, *P* = 0.001

^e^χ^2^ = 7.17, *P* = 0.007

^f^χ^2^ = 4.65, *P* = 0.031

^g^χ^2^ = 13.23, *P* = 0.000

^h^χ^2^ = 5.25, *P* = 0.022

We then performed logistic regression models to evaluate the influence of HLA-G polymorphisms on the susceptibility to HPV18-related cervical lesions (adjusted for age). No significant associations were observed when the HPV18 group was subdivided into CIN1 and CIN2+. Notably, the age of patients in the CIN2+ subgroup was much older than in the CIN1 subgroup (50.2 ± 12.3 vs. 40.5 ± 10.1), which shows that the HPV18 genotype does require a longer time period to become carcinogenic and lead to a progression from low-grade to high-grade cervical lesions.

### HLA-G expression association with the progression of HPV18-related cervical lesions

To investigate the association of HLA-G protein expression with HPV18-related cervical carcinogenesis, we analyzed 26, case-matched, HPV18-related, cervical tissues by IHC, with anti-HLA-G mAb 4H84 for HLA-G expression (2 ADC, 3 CIN3, 1 CIN2, 11 CIN1, and 9 normal tissues). We found that 66.7% (4/6) of CIN2+ lesions were classified as HLA-G positive, while only 18.2% (2/11) of CIN1 lesions were HLA-G positive. HLA-G staining yielded a negative result in normal tissues.

**Fig. 1:**
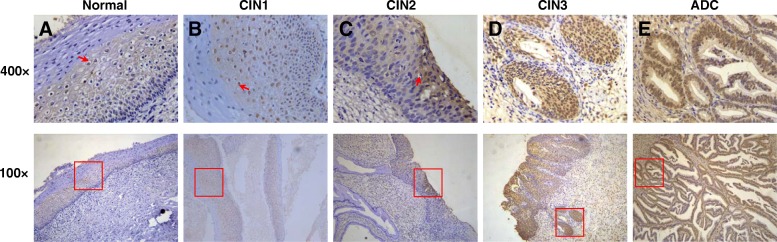
Immunohistochemical staining of HLA-G expression in cervical lesions. (**a**) normal cervical tissue. The arrow refer to HPV hollowed out cells; (**b**) CIN1 lesion. The arrow refer to basal cells of the epithelium; (**c**) CIN2 lesion; (**d**) CIN3 lesion. Squamous cell abnormalities involving glands; (**e**) Adenocarcinoma. HLA-G protein is diffusely expressed in cytoplasm and membrane. Positive for HLA-G expression increased from CIN1 to CIN2/3 lesions and was highest in ADC lesions

A strong and uniform HLA-G staining was observed in basal cells of the epithelium, where cells hollowed out by HPV were observed (Fig. 1A and B). HLA-G protein is diffusely expressed in cytoplasm and cell membrane. A higher frequency of HLA-G staining was observed in squamous epithelial cells among high-grade CIN lesions (Fig. 1C - E). Overall, positive for HLA-G expression increased from CIN1 to CIN2/3 lesions and was highest in patients with ADC; however, a significant association with the polymorphisms of HLA-G was not observed. The limited sample size of case-matched, HPV18-related, cervical tissues examined in this study may have influenced the results.

## Discussion

Persistent infection with oncogenic HPV types, especially HPV16 and HPV18, has a strong association with cervical cancer [15, 16]. This conclusion was also confirmed in our previous epidemiological study, where we found that the natural prevalence of HPV16 was 3.1% in the general population, but increased significantly in CIN2 (31.6%) and CIN3 (47.9%) lesions, reaching 60.6% in ICC. We observed a similar distribution pattern for HPV18. The natural prevalence of HPV18 was 1.5% in the general population, and increased steeply, to 5.6%, in CIN2+ lesions [11]. However, this is far lower than that the 13.0% figure reported in a global meta-analysis [17]. Moreover, our previous findings also indicated that HLA-G expression was associated with disease progression in cervical cancer [9]. Mounting evidence has shown that, besides oncogenic HPV types, other risk factors may be associated with the progression of cervical cancer, as well. These risk factors include aberrant expression of HLA-G [9, 18–20], polymorphisms of the HLA-G gene [10, 21–23], smoking habits and immune suppression [24].

HLA-G is a natural, tolerogenic molecule, with well-characterized immunoinhibitory properties. Its expression in cervical cancer cells may enable them to escape immunosurveillance [5, 9]. Our previous study revealed the involvement of the HLA-G molecule in the susceptibility to and outcome of HPV infections [10]. The HLA-G*01:01, −*01:03, −*01:04, −*01:06 and *14 bp In* alleles were associated with an increased risk of HPV infection acquisition and persistence, which may potentially impact the development of cervical lesions [10, 22, 23, 25–27]. The HLA-G *14 bp Del* and + 3142*C* alleles seems to confer protection against, instead of being a risk factor for, HPV infections [10, 22, 25–27]. The *14 bp In* allele in the *3′ UTR* of the HLA-G gene is associated with lower sHLA-G plasma levels [28–30]. However, the results obtained were inconclusive and even contradictory, as the HLA-G molecule was observed differentially at either decreased [31] or increased expression levels [9, 18, 19, 32] in cervical cancer tissues. Therefore, the level of HLA-G expression in malignant cells may vary according to the type of cancer. In the present study, we investigated the association of the HLA-G polymorphism and its expression with the development of cervical lesions caused by either HPV16 or HPV18. To the best of our knowledge, this study is the first to explore the potential relationship between the HLA-G molecule and HPV18-related cervical cancer risk. We observed that the HLA-G *14 bp In* and + 3142*G* alleles are risk factors for an HPV18 infection and influence the progression of HPV18-related cervical lesions. In addition, HLA-G protein expression increased from CIN1 to CIN2/3 lesions and was highest in patients with ADC; however, a significant association of these lesions with HLA-G polymorphisms was not observed. The limited sample size of case-matched, HPV18-related, cervical tissues examined in this study may have influenced the results.

Expression of the HLA-G protein at different stages and sites of cervical lesions is dependent on a combination of transcription factors, miRNAs, and microenvironmental factors. The aspects of the host’s genetic diversities that impact an immune response are likely determine those who are at higher risk for progressing to cervical cancer among infected individuals. The *14 bp In* and *del* alleles alter the set of miRNAs that are able to play a crucial regulatory role by targeting mRNAs for silencing. The +3142*C/G* polymorphism, located 167 bp downstream from the 14 bp polymorphic site, is also thought to influence miRNA binding. It was demonstrated that the +3142*G* allele may influence HLA-G expression by increasing its binding affinity for miRNAs (miR-148a, −148b and − 152), result in a downregulation of HLA-G protein expression through mRNA degradation and translation suppression [33–35]. The miR-mediated inhibition of HLA-G was shown to enhance NK cell recognition [34]. Furthermore, the *14 bp In/Del* and + 3142*C/G* polymorphisms are in a state of linkage disequilibrium [35, 36]; their combination into haplotypes may be involved in the regulation of HLA-G gene expression [36, 37]. In our population, three (*14 bp In/*+3142*G, 14 bp Del/*+3142*G, 14 bp Del/*+3142*C*) of four possible haplotypes (*14 bp In/*+3142*G, 14 bp Del/*+3142*G, 14 bp In/*+3142*C, 14 bp Del/*+3142*C*) were found (Table 2). Similar results were reported by Silva et al. [27] in Brazil and Ferguson et al. [22] in Canada, where a significantly increased cervical cancer risk was found to be associated with the *14 bp In/*+3142*G* haplotype. However, inconsistent results were obtained by Bortolotti et al. [21] in Italy and Yang et al. [38] in Taiwan, where a significantly decreased cancer risk was found to be associated with the *14 bp In/*+3142*G* haplotype. The discrepancy between the results of these studies and ours may be due to differing ethnicities, ages, sample sizes, genetic diversities, risk factors associated with lifestyle, and environmental factors.

In this study, we investigated the total HLA-G expression in HPV18-related cervical tissues with anti-HLA-G mAb 4H84, by IHC. We found that the HLA-G protein was detected in the basal cells of the epithelium, which is the initial site of HPV infections. A higher frequency of HLA-G staining was observed in squamous epithelial cells among high-grade CIN lesions. However, a significant association between lesions and the polymorphisms of HLA-G was not observed. The largest limitation of our study was sample size. Since the time between HPV infections and CIN3 lesions was calculated to be 7–15 years post-infection [39], this results in a limited sample size of case-matched, HPV18-related, cervical tissues in this study (which may have affected the outcome). Due to the small sample size, the statistical analyses involving the levels of HLA-G protein expression and six cases of HPV18-related high-grade cervical lesions, were hindered. Our data demonstrated that HLA-G expression progressively increased from CIN1 to CIN2/3 lesions and was highest in patients with malignant cervical lesions. These results were consistent with those of previous studies, which revealed that HLA-G is selectively expressed in cervical tissues, is associated with HPV infections [19, 31] and is associated with cervical lesion progression [9, 18, 32]. Guimarães et al. [31] reported that the HLA-G5 isoform (with mAb 5A6G7) was detected in 25 out of 74 cervical cancer tissues, but not in all HPV-related cases. Moreover, Dong et al. [19] found that HLA-G expression was significantly higher in CIN and cervical cancer lesions infected with HPV16/18 than in HPV-negative patients. In our study, all of the patients were infected with HPV18, and it is possible that HPV could contribute to HLA-G expression. Indeed, the mechanisms of HPV involvment in the modulation of HLA-G expression among different types of cervical cancer remain to be determined. This association may be influenced by the tumor microenvironment, as well as by the pathogenesis underlying the malignant transformation of the cells, such as transcriptional regulation, epigenetic modifications, and cytokine profiles.

## Conclusion

Our results support that the *HLA-G 14 bp In* and + 3142*G* alleles are risk factors for HPV18 infections and influence the progression of HPV18-related cervical lesions in the Chinese Han population. Our findings suggest that HLA-G-driven immune mechanisms play an important role in the progression from low-grade to high-grade lesions in cervical disease. However, the clinical relevance of HLA-G in cervical cancer needs to be further explored.
